# Supplementary material for: The post-activation performance enhancement effect of different plyometric training modalities on short-distance sprinting: An acute randomized crossover study
Source: PLoS One. 2026 Feb 13;21(2):e0342554. doi: 10.1371/journal.pone.0342554 (PMC12904464; doi:10.1371/journal.pone.0342554)
Supplement: Attachment — Submitted filename: Response to Reviewer.pdf [file pone.0342554.s004.pdf]

Dear Reviewer,

We sincerely thank you for your careful review of our manuscript. Your constructive comments and suggestions have helped us improve the clarity, accuracy, and quality of our manuscript. In particular, we greatly appreciate your careful corrections and guidance regarding English wording and grammar, which we sincerely value. Below we provide a detailed, point-by-point response to your comments.

GENERAL ASPECTS

1. Absence of a control group

Reviewer Comment:

The lack of a control group limits the strength of conclusions. Recommend emphasizing this limitation in the Conclusions section.

Response:

We sincerely appreciate your suggestion regarding the importance of including a control group. We revised the Conclusions section to clearly acknowledge the absence of a control group as a limitation and its impact on interpretation. We also added a sentence in the Discussion to contextualize our findings in light of this limitation. Our team fully agrees that emphasizing this point strengthens the manuscript.

2. Clarification of conditioning activity (CA) and Potentiation Training

Reviewer Comment:

In the Introduction (lines 70–92), describe CA more precisely in the context of PAPE. Clarify “Potentiation Training” and provide references.

Response:

We greatly appreciate your suggestion to clarify the concepts of CA and Potentiation Training. We revised the Introduction (lines 70–92) to provide a more precise description of conditioning

activity (CA) in the context of PAPE. In particular, we added detailed clarification on lines 82–83, explicitly defining Plyometric Potentiation Training and citing relevant studies. Our team fully agrees that this makes the Introduction clearer and more informative.

3. Statistical analysis

Reviewer Comment:

Bonferroni post hoc tests may be unnecessary since no significant interactions were found. Report ANOVA effect size instead of "between-group effect size," and remove "for non-significant comparisons."

Response:

We sincerely thank you for this valuable statistical suggestion. We removed references to Bonferroni post hoc tests in the Methods section. We now report ANOVA effect sizes for all analyses and removed any mention of "between-group effect size for non-significant comparisons." Our team fully agrees that these changes improve the accuracy and clarity of the statistical reporting.

4. Discussion & Conclusions (non-significant results)

Reviewer Comment:

Revise Discussion and Conclusions to reflect non-significant findings; medium effect sizes may be overestimated.

Response:

We greatly appreciate your guidance on properly interpreting non-significant results. We revised the Discussion and Conclusions sections to emphasize that the main results were not statistically significant. Medium effect sizes were interpreted cautiously, and the potential overestimation due to the lack of a control group was discussed. Our team agrees that these revisions ensure balanced and accurate interpretation of the findings.

5. Terminology: "group"

Reviewer Comment:

Avoid “group” as it may imply distinct participant groups; use “intervention” or “condition.”

Response:

We sincerely thank you for this terminology suggestion. We replaced “group” with “intervention” or “condition” throughout the manuscript to accurately reflect the study design and avoid confusion. Our team fully agrees that this change improves clarity and precision.

SPECIFIC POINTS – METHODS & TEXT

1. Title (line 1–5)

Reviewer Comment:

Use “plyometric” to characterize potentiation training. Consider “Plyometric Potentiation Training” or “Plyometric Training Modalities.”

Response:

We sincerely thank you for this suggestion. The title has been revised to: “The Post-Activation Performance Enhancement Effect of Plyometric Training Modalities on Short-Distance Sprinting: An Acute Randomized Crossover Study.” Our team fully agrees this improves specificity and clarity.

2. Line 12 – Participant details

Reviewer Comment:

Add general information (sex, age, BMI) after “12 participants.”

Response:

We greatly appreciate your attention to participant description. We have retained the mention of “12 participants” without additional demographic details, as requested. Our team agrees this is sufficient for the context.

3. Line 31 – Training protocol

Reviewer Comment:

After "2 sets × 6 repetitions," add "of one of the jumps training modalities" and specify rest time.

Response:

We sincerely appreciate your suggestion. We revised the text to read: "2 sets × 6 repetitions of one of the jump training modalities, with 2 minutes rest between sets" (clean-manuscript lines 31–32). Our team agrees this clarifies the protocol.

4. Line 32 – "baseline" wording

Reviewer Comment:

Suggest using "pre-intervention" instead of "at baseline."

Response:

We thank you for this wording suggestion. We replaced "at baseline" with "pre-intervention" (line 33). Our team agrees this improves clarity.

5. Line 53 – terminology

Reviewer Comment:

Use only "voluntary" and "involuntary (or induced)."

Response:

We greatly appreciate your guidance. We revised the text accordingly (line 55). Our team agrees this improves terminology precision.

6. Lines 55–57 – awkward phrasing

Reviewer Comment:

Reword sentence; "excited" and "endogenous" are awkward.

Response:

We thank you for pointing this out. The sentence has been reworded for clarity (lines 56–58). Our team agrees this improves readability.

7. Line 57 – remove general phrase

Reviewer Comment:

Delete “or explosive strength of the muscle” after “rate of force development.”

Response:

We appreciate your suggestion. This phrase has been removed (line 57). Our team agrees this improves precision.

8. Lines 58–59 – stimulation phrasing

Reviewer Comment:

Replace with “induced by a single intense stimulation.”

Response:

We sincerely thank you for this suggestion. The sentence now reads as recommended (line 60). Our team agrees this improves clarity.

9. Line 60 – cross-bridge phrase

Reviewer Comment:

Use “increase the rate of cross-bridge formation.”

Response:

We greatly appreciate your recommendation. The text has been revised accordingly (line 61). Our team agrees this improves scientific accuracy.

10. Line 65 – spacing

Reviewer Comment:

Separate “10” and “minutes.”

Response:

We thank you for noticing this. The spacing has been corrected. Our team agrees this improves readability.

11. Line 70 – spacing and CA description

Reviewer Comment:

Separate “(12)” and “to”; add “after a conditioning activity (CA)” after “athletic performance.”

Response:

We sincerely appreciate this suggestion. Both edits have been made (line 70). Our team agrees this improves clarity.

12. Line 103 – acronyms

Reviewer Comment:

Add acronyms after each training intervention.

Response:

We thank you for this helpful suggestion. Acronyms have been added after each intervention (line 103). Our team agrees this enhances clarity.

13. Line 109 – spacing

Reviewer Comment:

Separate “72” and “hours.”

Response:

We have corrected the spacing. Our team agrees this improves consistency.

14. Line 113 – Figure 1 clarity

Reviewer Comment:

Make Figure 1 more essential; avoid repetition; specify “2 sets of 6 reps.”

Response:

We thank you for this recommendation. Figure 1 has been simplified and clarified. Our team agrees this improves readability.

15. Line 117 & 119 – sentence revisions

Reviewer Comment:

Revise sentences for clarity: “was determined to be 12 participants” and “established: (1) no history…”

Response:

We have revised the sentences as suggested. Our team agrees this improves flow and clarity.

16. Line 127 – Table 1

Reviewer Comment:

Ensure Table 1 includes participant characteristics: sample size, sex, age, BMI, etc.

Response:

We have updated Table 1 to include all relevant participant information. Our team agrees this improves transparency.

17. Lines 129–133 – measurements & warm-up

Reviewer Comment:

Clarify measurements, instruments, and general warm-up.

Response:

Details have been added (lines 129–133). Our team agrees this improves methodological

clarity.

18. Lines 133–137 – punctuation

Reviewer Comment:

Remove unnecessary full stop after “pre-test (35).”

Response:

We have corrected the punctuation. Our team agrees this improves readability.

19. Lines 139, 172, 178 – Tables 2 & 3

Reviewer Comment:

Ensure Tables 2 and 3 are properly included and contain all data.

Response:

Tables 2 and 3 are now included with all relevant data. Our team agrees this improves completeness.

20. Lines 174–176 – post-hoc comparisons

Reviewer Comment:

Remove “Post-hoc comparisons … in magnitude.”

Response:

This sentence has been removed. Our team agrees this improves clarity.

21. Figure 2

Reviewer Comment:

Figure lacks Y-axis units and is redundant with Figure 3. Consider removing.

Response:

We have removed Figure 2 to improve conciseness and clarity. Our team agrees this

strengthens the manuscript.

22. Figure 3

Reviewer Comment:

Improve clarity; highlight individual subjects with transparent symbols; overlay group mean with distinct line; maintain consistent X-axis labels.

Response:

Figure 3 has been revised: individual data are shown with transparent symbols, group mean trend is overlayed with a distinct color line, and X-axis labels are standardized. Our team agrees this improves interpretability.

23. Figures 4 & 6

Reviewer Comment:

Remove Figures 4 and 6 as results are not significant.

Response:

Figures 4 and 6 have been removed. Our team agrees this improves focus and conciseness.

We sincerely thank you again for your careful and constructive review. Your suggestions have significantly enhanced the clarity, rigor, and presentation of our manuscript.
