# Supplementary material for: The post-activation performance enhancement effect of different plyometric training modalities on short-distance sprinting: An acute randomized crossover study
Source: PLoS One. 2026 Feb 13;21(2):e0342554. doi: 10.1371/journal.pone.0342554 (PMC12904464; doi:10.1371/journal.pone.0342554)
Supplement: Attachment — Submitted filename: Response_to_Reviewer_auresp_3.pdf [file pone.0342554.s005.pdf]

We sincerely thank the reviewer for the thorough evaluation of our manuscript and for the constructive and insightful comments, as well as for the considerable time and effort devoted to improving the quality of our work. We are especially grateful for the generous and selfless guidance regarding our English writing. As English is not our first language, unintentional issues and oversights inevitably occurred during the manuscript preparation process. We truly appreciate the reviewer's patience, understanding, and thoughtful suggestions, which are of great value to us and will serve as strong motivation for our future academic work, extending well beyond the present manuscript.

Below, we provide a numbered, point-by-point response to all comments. All revisions have been carefully addressed and incorporated into the revised manuscript.

# GENERAL ASPECT

1. Spacing and punctuation consistency

Please revise the manuscript carefully with particular attention to spacing and punctuation. In several sections, spacing is inconsistent, which affects readability and professionalism.

Response:

The entire manuscript has been carefully proofread, and all issues related to spacing, punctuation, and formatting inconsistencies (e.g., missing spaces after commas or before parentheses) have been corrected. A comprehensive formal language review was conducted to ensure consistency, readability, and professional presentation throughout the manuscript.

# SPECIFIC POINTS

2. Title (line 1-5): I suggest the authors evaluate the use of the world “different” before “Pliometric Training Modalities”. The title should sound like “The Post-Activation Performance Enhancement Effect of Different Plyometric Training Modalities on Short-Distance Sprinting: An Acute Randomized Crossover Study”

Response:

The title has been revised to:

“ The Post-Activation Performance Enhancement Effect of Different Plyometric Training Modalities on Short-Distance Sprinting: An Acute Randomized Crossover Study.”

3. Line 27: I suggest using “CJ -PT” instead of “MX-PT”, to identify “combined jump plyometric training”.

Response:

The abbreviation MX-PT has been replaced with CJ-PT (combined jump plyometric training) throughout the entire manuscript, including the text, tables, and figures.

4. Line 41: I suggest using “short-term effects of different plyometric based conditioning activities” instead of “short-term effects of conditioning activities”.

Response:

The sentence has been revised accordingly to explicitly specify “different plyometric-based conditioning activities,” improving clarity and precision.

5. Line 50: Instead of “excitation”, please consider re-writing the sentence using “potentiation”.

Response:

The term “excitation” has been replaced with “potentiation”.

6. Line 53-54: I recommend revising this sentence for clarity and readability. Instead of “the neuromuscular system is activated endogenously through maximal or near-maximal voluntary contractions, resulting in an acute increase in the rate of force development,” you may consider using the following formulation:

“…the neuromuscular system performance is enhanced through maximal or near-maximal voluntary contractions, resulting in an acute increase in force production and in the rate of force development.”

Response:

The sentence has been revised exactly as suggested to enhance clarity, readability, and physiological accuracy.

7. To improve clarity, you might consider rephrasing it as follows : “Over the years, this

principle has become a classic component of sports training and has been increasingly translated into practical applications within sport performance settings.”

Response:

The sentence has been rephrased as recommended, improving flow .

8. Line 97-98: This sentence could be improved for clarity and readability, I suggest “All participants completed two familiarization sessions to ensure they were fully comfortable with the sprint tests and the various plyometric exercises before the experimental sessions.”

Response:

The sentence has been revised exactly as suggested to clearly describe the familiarization process.

9. Line 104: please consider adding “and fatigue” after “residual training”.

Response:

The phrase has been updated to “residual training and fatigue.”

10. Line 112: I suggest using “:” after “established”

Response:

The punctuation has been corrected as suggested.

11. In the paragraph “Post-activation performance enhancement protocols”, please clarify the structure of the combined jump plyometric training (which I recommend referring to as CJ-PT for consistency). Specifically, it is not entirely clear whether one repetition consists of a tuck jump immediately followed by a horizontal frog jump. If this is the case, please state it explicitly to ensure the protocol is fully understandable and replicable. Additionally, you may consider revising the title of this paragraph to better reflect its content. For example, “Plyometric Conditioning Activity Protocols” might provide a clearer and more accurate description.

Response:

The CJ-PT protocol has been clarified by explicitly stating that one repetition consisted of a tuck jump immediately followed by a horizontal frog jump, ensuring transparency and

replicability (see manuscript lines 143–146 for details).

The paragraph title has been revised to “Plyometric Conditioning Activity Protocols.”

12. Line 151: Please define the acronym ANOVA the first time it appears in the manuscript. Even though it is a well-known statistical term, it is good practice to write it out in full before introducing the abbreviation. In this case, you may use: “Analysis of Variance (ANOVA)”.

Response:

ANOVA has been defined at first mention as “Analysis of Variance (ANOVA).”

13. Line 152-153: I recommend revising this sentence for clarity. Instead of “Between-intervention effect sizes (ES) were reported as $\eta p^2$, and within-intervention ES as Cohen’s d (41)”, you may consider using the following formulation:

“The ANOVA effect sizes (ES) were reported as $\eta p^2$, and for pairwise comparisons Cohen’s d (41) was reported.”

Response:

We have revised the sentences as suggested. Our team agrees this improves flow and clarity.

14. Regarding Figure 1, I have a few suggestions to improve clarity and consistency:

1. Please align the "SESSION 1" box with the other session boxes for visual consistency. If "SESSION 1" refers to the two familiarization sessions, I recommend specifying them as “FAMILIARIZATION 1” and “FAMILIARIZATION 2” to avoid confusion.

2. In the PAPE box, I suggest adding a space for readability: “PAPE 2 sets of 6 reps (Self respect)” instead of “PAPE 2 sets of 6 reps(Self respect)”.

3. Please clarify the meaning of “Self respect” in this context, as it is unclear to the reader.

In Table 1, please adjust the formatting by placing the closing parenthesis immediately after “years” to ensure consistency and correct punctuation.

In Table 2, please clarify the meaning of “self respect”, as its interpretation within the context of the protocol is not evident. Additionally, the term “intergroup interval” requires clarification: does it refer to the recovery between repetitions or between sets? Please specify this to avoid ambiguity. In the first column, I suggest replacing the term “Group” with “CA”, “Conditioning

Activity", or "Plyometric CA", as these alternatives provide a more accurate and descriptive label for the conditioning stimulus used. I also remind you to consider replacing MX-PT with CJ-PT, as previously recommended, to ensure consistent terminology throughout the manuscript.

In Figure 2, please include the unit of measurement on the y-axis. In this case, time (s) should be specified to ensure clarity and consistency with standard reporting practices.

In Figure 2 and Table 3, please remember to consider replacing MX-PT with the revised terminology previously suggested (e.g., CJ-PT), to maintain consistency throughout the manuscript.

In Figure 3, please ensure consistency in the symbols used to represent the horizontal vs. mixed (which I recommend renaming to combined) conditions. At 4, 8, and 16 minutes you used a diamond symbol, whereas at 12 minutes a circle appears. This inconsistency may confuse readers, so I recommend standardizing the symbol across all time points.

Response:

Figures 1–3 and Tables 1–3 have been revised according to your suggestions, and we sincerely thank you for your valuable advice.

# Final Statement

We sincerely thank the reviewer for the insightful comments and constructive suggestions. We believe that these revisions have substantially improved the clarity, rigor, and overall quality of the manuscript.
