# Supplementary material for: The post-activation performance enhancement effect of different plyometric training modalities on short-distance sprinting: An acute randomized crossover study
Source: PLoS One. 2026 Feb 13;21(2):e0342554. doi: 10.1371/journal.pone.0342554 (PMC12904464; doi:10.1371/journal.pone.0342554)
Supplement: Attachment — Submitted filename: Response_to_Reviewer_auresp_4.pdf [file pone.0342554.s006.pdf]

Response to Reviewer #2

We sincerely thank Reviewer #2 for the positive evaluation of our manuscript and for the constructive comments, which have helped us further improve the clarity, consistency, and presentation quality of the paper. We have carefully addressed all the points raised. Our detailed responses are provided below.

General Comment

Reviewer’s comment:

I appreciate the effort you have made to revise the manuscript according to the previous feedback. The paper is now clearer and more structured. However, a few minor aspects can still be improved, particularly regarding the graphical presentation of the figures and the formal presentation of the text.

Response:

We thank the reviewer for this positive assessment. Following the reviewer’s suggestions, we have carefully revised the manuscript to improve formatting consistency, correct typographical issues, and enhance the clarity and visual presentation of the figures.

Specific Points

1. Formatting of time points at line 33

Reviewer’s comment:

There is a lack of spacing between the time points in the sentence describing the 5-meter sprint performance assessments.

Response:

Thank you for pointing this out. We have revised the sentence at line 33 to include appropriate spacing between the time points for improved readability. The sentence now reads:

“5-meter sprint performance pre-intervention and at 4, 8, 12, and 16 minutes post-training.”

2. Conclusion of the Abstract – sample size and sex differences

Reviewer’s comment:

Given that one of the main limitations of the present study is the sample size, future studies should explicitly include a larger sample and investigate responses in both sexes.

Response:

We agree with this important suggestion. The Conclusion of the Abstract has been revised to explicitly acknowledge the limitation related to sample size and to emphasize that future studies should include a larger sample and further investigate potential sex-specific responses, in addition to controlling confounding factors and using surface electromyography.

3. Spacing and formatting corrections (lines 85, 115, 137, 206, 209, 216, 218)

Reviewer’s comment:

Please insert missing spaces between words, numbers, units, and references at the specified lines and verify whether similar spacing issues occur elsewhere in the manuscript.

Response:

We thank the reviewer for highlighting these formatting issues. All the indicated spacing errors at lines 85, 115, 137, 206, 209, 216, and 218 have been corrected. In addition, we carefully re-checked the entire manuscript and corrected any similar spacing inconsistencies to ensure uniform formatting throughout the text.

4. Extra parenthesis at line 242

Reviewer’s comment:

There is an extra opening parenthesis at line 242.

Response:

Thank you for noticing this error. The extra parenthesis at line 242 has been removed to ensure correct punctuation and formatting.

5. Missing period at line 251

Reviewer’s comment:

Please insert a period before “Future” to correctly separate the sentences.

Response:

This has been corrected. A period has been added before “Future” at line 251 to properly separate the sentences.

6. Inconsistency between Study Design and Figure 1 (72 h vs. 48 h)

Reviewer’s comment:

There is an inconsistency between the text (minimum 72 hours between sessions) and Figure 1 (48-hour interval).

Response:

We appreciate the reviewer bringing this inconsistency to our attention. The manuscript and Figure 1 have now been revised to ensure consistency. The correct interval between intervention sessions is 72 hours, and Figure 1 has been updated accordingly to match the description in the Study Design section.

Comments on Figures

7. Figure 1 – “Session 1” and familiarization sessions

Reviewer’s comment:

If “Session 1” refers to the two familiarization sessions, it should be removed, retaining only “Familiarization 1” and “Familiarization 2”.

Response:

We agree with this suggestion. “Session 1” has been removed from Figure 1, and the two familiarization sessions are now clearly presented as “Familiarization 1” and “Familiarization 2” in separate boxes. The remaining sessions have been renumbered accordingly to improve clarity.

8. Figure 1 – terminology in the PAPE section

Reviewer’s comment:

Use VJ-PT, HJ-PT, and CJ-PT instead of “Process 1”, “Process 2”, and “Process 3”.

Response:

Thank you for this helpful recommendation. The labels in the PAPE section of Figure 1 have been revised to VJ-PT, HJ-PT, and CJ-PT to ensure consistency with the terminology used throughout the manuscript.

9. Figure 3 – spacing between time points and the box

Reviewer’s comment:

The spacing between time points (16 min, 12 min, 8 min, 4 min) and the box is inconsistent and affects visual balance.

Response:

We agree with the reviewer’s observation. Figure 3 has been revised to ensure consistent spacing between all time points and the box, thereby improving the visual balance and overall clarity of the figure.

Final Statement

We sincerely thank the reviewer for the insightful comments and constructive suggestions. We believe that these revisions have substantially improved the clarity, rigor, and overall quality of the manuscript.
