# Supplementary material for: The post-activation performance enhancement effect of different plyometric training modalities on short-distance sprinting: An acute randomized crossover study
Source: PLoS One. 2026 Feb 13;21(2):e0342554. doi: 10.1371/journal.pone.0342554 (PMC12904464; doi:10.1371/journal.pone.0342554)
Supplement: Attachment — Submitted filename: Response to Reviewers.docx [file pone.0342554.s003.docx]

Dear Reviewer

Thank you for your positive appraisal of our study and for the reviewers’ thorough review and constructive feedback; on behalf of all co-authors, I thank you for granting us the opportunity to revise the manuscript. We have carefully considered these comments and made the necessary corrections in the hope of enhancing the coherence and clarity of the study. In our response, line numbers correspond to the revised manuscript, and we also provide a clean version to facilitate readability. The principal modifications and our replies to the reviewers’ comments are presented below.

1: General Evaluation

The article addresses a timely and practically relevant topic in sports science — the comparison of the effectiveness of three types of plyometric potentiation training (VJ-PT, HJ-PT, MX-PT) in inducing the post-activation performance enhancement (PAPE) effect in the context of a 5-meter sprint. The study is properly designed and includes detailed analysis, but it also has several limitations that require consideration.

Response to Comment 1：Thank you for your positive appraisal of our study and for the reviewers’ thorough review and constructive feedback; on behalf of all co-authors, I thank you for granting us the opportunity to revise the manuscript. We have carefully considered these comments and made the necessary corrections in the hope of enhancing the coherence and clarity of the study. In our response, line numbers correspond to the revised manuscript, and we also provide a clean version to facilitate readability. The principal modifications and our replies to the reviewers’ comments are presented below.

2. Strengths

Appropriately selected study design (randomized crossover)

Use of dynamic warm-up and control of procedural variables

Practical implications for coaches in speed-based sports

Use of Cohen’s d for interpreting effect sizes

Cautious language in the conclusions ("potential effect", "suggested peak")

Response to Comment 2：Thank you for your meticulous review; we sincerely appreciate your affirmation of our work, and your precise and professional comments have been deeply encouraging. We will continue to uphold a rigorous and pragmatic attitude, striving to deliver even more satisfactory results in future endeavors. At the same time, we remain committed to advancing the field on the foundation laid by our predecessors, in gratitude for your recognition.

3. Limitations and Suggestions

3.1. Lack of a control group:

Response to Comment 3.1：Thank you for the recommendation: “Consider addressing this point in the ‘Limitations’ section and, where feasible, include a control group in future studies. This would help clarify whether PAPE is the result of a specific intervention or may arise spontaneously.” We have adopted your suggestion with gratitude; your candid advice is truly inspiring, and we have added the corresponding statement in the Limitations section (clean manuscript, lines 258–259).

3. 2. Lack of information about participants' sport disciplines:

Response to Comment 3.1：Indeed, we recognize that PAPE responses differ markedly across individuals of varying training levels and sport-specific demands; at recruitment, our third criterion—“regular training participation 3–5 times per week”—implicitly encompassed both speed- and strength-oriented routines but lacked explicit sport-specificity. Accordingly, we have now clarified this point in the third recruitment criterion and in the Limitations section (clean manuscript, lines 259–261).

3. 3. Potential fatigue effects from four sprints in one session:

Response to Comment 3.3：Thank you for this valuable suggestion. We acknowledge the issue you raised: when designing the study we relied on previous findings regarding the time course of PAPE, yet we now recognize that repeated testing can induce cumulative fatigue and allow participants to improve merely by becoming familiar with the protocol. We have therefore added this limitation to the manuscript (clean-Manuscript, lines 263–264) and again express our sincere gratitude for your insightful feedback.

3.4. Inconsistency between assumptions and HJ-PT results:

Response to Comment 3.4：Upon re-examining our manuscript, we sincerely apologize for the translation errors identified. We have now amended the text accordingly (clean-Manuscript, lines 204–215), as follows: “In short-distance acceleration, net horizontal force determines the magnitude of acceleration(44–47). Previous research indicates that elite sprinters generate greater horizontal impulse during the acceleration phase, resulting in higher acceleration (48). Similarly, training protocols that exhibit a higher degree of similarity to the movement patterns during exercise have been shown to produce greater transfer effects in terms of explosive power improvements compared to non-specific movement patterns. (45). However, during sprinting, mechanical variables are entangled, and no single variable is associated with better performance(49) This may account for the absence of an immediate sprint performance benefit following HJ-PT. Moreover, horizontal-jump drills typically involve ground-contact times exceeding 100 ms(50), which are markedly longer than the stance phase of sprinting (~80–90 ms)(49); this discrepancy may further magnify measurement bias in the PAPE response. ”

3.5. Small sample size (n = 12):

While statistically acceptable as a minimum, this sample size limits the detection of subtle effects and reduces generalizability.

Response to Comment 3.5：We have revised the Limitations section as suggested; please refer to clean-Manuscript, lines 258–260.

3.6. No physiological measurements:

Response to Comment 3.6：Indeed, our team has conducted a more in-depth examination of the physiological indices and have incorporated a statement to this effect in the Limitations section; please see clean-Manuscript, lines 266–269.

3.7. Potential learning effect:

Response to Comment 3.7：As outlined in Section 3.3, we have implemented the requested revisions; please refer to clean-Manuscript, lines 263–264.

3.8. Use of only 5-meter sprint:

Response to Comment 3.8：Indeed, during the initial design phase, our survey indicated that shorter sprint distances yield a stronger correlation with peak power output; moreover, owing to the intertwined kinetic and kinematic constraints of sprinting, athletes cannot readily exploit capacities they do not inherently possess. We therefore set the test distance at 5 m and have clarified this rationale in the Test Procedures section. To broaden the scope of future investigations, we have also added a recommendation in the Limitations section for the inclusion of 10–20 m sprints to afford a more comprehensive assessment of acceleration performance (see clean-Manuscript, lines 148–149 and 265).

4. Data Presentation

The data are generally well-presented, but:

There is no graphical display of individual participant results (e.g., spaghetti plots)

Percent change from baseline is not shown

Statistical significance is not marked on graphs (e.g., *, **)

Suggestion: Improve data visualization by including individual trends, percentage changes, and markers for statistical significance.

Response to Comment 4 We have incorporated the requested revisions and added new Figures 2 and 3 accordingly.

5. Evaluation of Conclusions

The conclusions are mostly consistent with the data, but:

The lack of statistical significance (p > 0.05) should be explicitly stated

The conclusions regarding HJ-PT are somewhat overstated — effects were minimal or negative

The explanation that MX-PT was ineffective due to fatigue should be presented as a hypothesis, not a fact

Response to Comment 5：Thank you for your suggestion; we have revised the Conclusions section accordingly, as follows: “By comparing the acute effects of vertical jump, horizontal jump, and MX‑PT on short‑distance sprint performance, vertical jump demonstrated potential advantages as pre‑competition activation strategies, although these differences were not statistically significant(p> 0.05). However, the complexity of MX‑PT movements led to fatigue accumulation that attenuated its efficacy. Furthermore, the vertical jump group appeared to exhibit peak PAPE effects between 4 and 8 minutes. It is recommended that potentiation exercise selections align movement patterns with sport‑specific demands while avoiding complex actions that provoke excessive fatigue. ”

6. Final Comment

It is recommended to refine the “Limitations” section, expand the description of participant backgrounds, clarify the interpretation of HJ-PT outcomes, and improve the graphical presentation of results. The study has both scientific and practical value, especially for coaches and researchers focused on PAPE and short-distance sprint performance.

Response to Comment 6：Thank you for your suggestion. As mentioned before, we have made the modifications.

Thank you for your suggestion; as previously stated, we have made the corresponding revisions. We once again express our heartfelt gratitude to each reviewer for the time invested and the invaluable comments provided. Guided by all recommendations, we have comprehensively revised the manuscript and believe that these changes have markedly improved its clarity, rigor, and overall quality. We trust that the revised version now meets your expectations and respectfully hope it will meet with your approval.

Kind regards,

Duanying li
